# Statin suppresses sirtuin 6 through miR-495, increasing FoxO1-dependent hepatic gluconeogenesis

**DOI:** 10.7150/thno.49770

**Published:** 2020-09-15

**Authors:** Min Yan Shi, In Hyuk Bang, Chang Yeob Han, Dae Ho Lee, Byung-Hyun Park, Eun Ju Bae

**Affiliations:** 1 Department of Biochemistry and Molecular Biology, Chonbuk National University Medical School, Jeonju, Jeonbuk 54896, Republic of Korea.; 2 College of Pharmacy, Chonbuk National University, Jeonju, Jeonbuk 54896, Republic of Korea.; 3 Department of Internal Medicine, Gil Medical Center, Gachon University College of Medicine, Incheon 21565, Republic of Korea.

**Keywords:** Sirt6, statin, gluconeogenesis, miR-495, FoxO1

## Abstract

**Rationale:** Statin, the most widely used medication in lowering cholesterol, is also associated with increased risk of type 2 diabetes, but its molecular basis remains unclear.

**Methods:** Mice were injected intraperitoneally with statins alone or in combination with sirtuin (Sirt) 6 activator, and blood glucose levels were measured. Liver tissues from patients with statin use were analyzed for the expression of Sirt6.

**Results:** Statin treatment up-regulated the hepatic expression of phosphoenolpyruvate carboxykinase and glucose-6-phosphatase, which was prevented by Sirt6 overexpression. Mechanistically, statin directly repressed Sirt6 expression by induction of microRNA (miR)-495, a novel inhibitor of Sirt6. Pathway analysis for predicted target genes of miR-495 recognized forkhead box protein (Fox)O1 as a key downstream signaling of Sirt6. Statin treatment increased the acetylation and protein stability of FoxO1, which was suppressed by Sirt6 overexpression. Inhibiting miR-495 recovered Sirt6 levels, blocking the ability of statin to increase FoxO1 mediated gluconeogenesis, and thus confirming the role of the miR-495/Sirt6/FoxO1 pathway in controlling gluconeogenesis. Moreover, the Sirt6 activator MDL801 prevented gluconeogenesis and hyperglycemia induced by statin in mice. Equally noteworthy was that human liver tissues obtained from statin users showed a significant decrease in Sirt6 protein levels compared to those of non-users.

**Conclusion:** Statin induces miR-495 to suppress Sirt6 expression, which leads to enhancement of FoxO1-mediated hepatic gluconeogenesis. Thus, Sirt6 activation may offer a promising strategy for preventing statin-induced hyperglycemia.

## Introduction

Statins are the most widely-prescribed medications for reducing blood cholesterol and the prevention of major cardiovascular events by inhibiting 3-hydroxy-3-methylglutaryl-CoA (HMG-CoA) reductase. Although generally recognized as safe and well tolerated, its adverse effects, including myopathy, are common among users and may significantly impact adherence to pharmacotherapy 1, 2. Recently, accumulating evidence from randomized controlled trials and meta-analysis have indicated a further and significant side effect: the risk of new-onset type 2 diabetes 3-7, requiring a safety warning in statin therapy. Meta-analysis reports indicate that statin-induced diabetes risk is dose-dependent, irrespective of its chemical structure (hydrophobicity) 6, 8. An intensive-dose regimen has a higher risk of the patient developing diabetes compared to a moderate-dose group 8. Moreover, the risk of developing diabetes with statin use was particularly pronounced in elderly patients 6.

Statin acts on multiple tissues, among which the liver, where cholesterol is synthesized, is one of the main organs impacted. Hepatic gluconeogenesis has been suggested as a primary pathogenesis for statin-induced glucose intolerance 9, 10. However, the precise mechanism by which increased gluconeogenesis is induced by statins has been little studied to date. Thus, identifying the molecular basis of hepatic insulin resistance with statin and preventive measures for this is urgent since discontinuation of statin significantly increases the incidence of cardiovascular events 1, 2. In light of evidence that age is the primary risk factor in statin-induced diabetes, we hypothesized that aging-related molecules may be involved in glucose dysregulation caused by statin.

Sirtuins are highly conserved NAD^+^-dependent protein deacetylases that have attracted attention owing to their activity in slowing the aging process and related forms of pathogenesis. Among the seven members of this family in mammals, Sirt6, has been suggested as showing more promise than other sirtuin members in extending longevity 11. Along with others, we have shown that Sirt6 protects against systemic insulin resistance and alcoholic- and nonalcoholic-fatty liver diseases by targeting multiple molecules 12-15. Abundantly expressed in the liver, Sirt6 has been reported to repress hepatic gluconeogenesis, which appears to occur via two main pathways: (i) deacetylation and activation of histone acetyltransferase GCN5, thereby increasing the activity of peroxisome proliferator-activated receptor gamma coactivator 1-alpha (PGC1α), a master co-activator of gluconeogenesis genes 16; and (ii) deacetylation and suppression of forkhead box protein (Fox)O1, a key transcription factor that activates phosphoenolpyruvate carboxykinase (PEPCK) and glucose-6-phosphatase (G6Pase) 17.

This study was designed to investigate the acute effect of statins on glycemia and its molecular mechanism associated with Sirt6, excluding confounding factors such as lowered blood cholesterol with chronic statin treatment. Our results demonstrate that statin treatment represses Sirt6 expression in the liver of mice and humans, thereby increasing FoxO1 transactivation and gluconeogenic gene expression. Expanding on earlier reports that statin controls various microRNAs (miRNAs) to impact glucose homeostasis, we also identify a novel miRNA increased by statins for direct regulation of Sirt6 expression.

## Methods

### Human tissues

Human liver tissues were obtained from the Gachon University Gil Medical Center from obese subjects (4 statin users and 4 non-users) during bariatric surgery. Those subjects were attending one of clinical studies that aimed to evaluate the performance of imaging studies on nonalcoholic fatty liver disease. The clinical and biochemical parameters of study participants were shown in Table S1. Study protocols were in accordance with the Declaration of Helsinki and were approved by the Institutional Review Board at the Gachon University Gil Medical Center (Approval No. KCT0003144 and KCT0003527). All participants provided written informed consent and the studies were registered at https://cris.nih.go.kr in accordance with the WHO International Clinical Trials Registry Platform.

### Animals and drug treatment

Eight- to ten-week-old C57BL/6 male mice (Damul Science, Daejeon, Korea) were maintained on a diet of standard laboratory chow and water *ad libitum* in a controlled barrier facility (12 h light/dark cycle, 23 ± 1 °C, 60-70% humidity). Mice were injected intraperitoneally with statins (10 or 30 mg/kg body weight in 10% DMSO in saline) or vehicle once a day for three consecutive days. Fed blood glucose level was measured 9 h after second administration of statins to mice. Blood glucose level was measured again 2 h after the last dosage of statin (total 17 h fasting). Reagents were purchased as follows; simvastatin, atorvastatin, and fluvastatin were from TCI (Portland, OR, USA), rosuvastatin and lovastatin were from Cayman Chemical (Ann Arbor, MI, USA), pravastatin, fucoidan, geranylgeranyl pyrophosphate (GGPP), farnesyl pyrophosphate (FPP), cholesterol, and 25-hydroxycholesterol were from Sigma-Aldrich (St. Louis, MO, USA), and MDL801 was from ChemScience (St-Laurent, QC, Canada). All animal experiments were performed in compliance with the *Guide for the Care and Use of Laboratory Animals*, published by the US National Institutes of Health (NIH Publication No. 85-23, revised 2011). The current study protocol was also approved by the Institutional Animal Care and Use Committee of Chonbuk National University (Approval No. CBNU-2019-0122).

### Preparation of recombinant adenovirus

Adenoviruses expressing Sirt6 (Ad-Sirt6), a catalytically inactive mutant Sirt6-H133Y (Ad-mSirt6), and β-galactosidase (Ad-LacZ) were prepared as described previously 13. Adenovirus (1 × 10^9^ pfu) was injected into mice via tail vein 3 days prior to statin administration.

### Bioluminescence imaging of liver

Adenovirus expressing G6Pase (-231/+57) luciferase (Ad-G6Pase-Luc) was kindly provided by Dr. Um SH (Sungkyunkwan University, Suwon, Korea). Ad-G6Pase-Luc (1 × 10^9^ pfu/kg of body weight) was injected into tail veins of mice. The following day, Sirt6 adenoviruses were intravenously injected into mice, followed by statin treatment for 3 consecutive days. After the last dosing of statin, 6 h-fasted mice were injected with 150 mg/kg of firefly D‐luciferin (GoldBio, St Louis, MO, USA). After 10 min, mice were anesthetized and imaged using the IVIS Luminar XR Imaging System (Caliper Life Sciences, Hopkinton, MA, USA).

### Cell culture and transient transfection

For isolation of primary hepatocytes from mice, livers were perfused with collagenase type IV (Sigma-Aldrich), and hepatocytes were prepared as described previously 18. The human hepatocarcinoma cell line HepG2 and murine hepatocyte cell line AML12 were obtained from the American Type Culture Collection (Manassas, VA, USA). HepG2 cells were maintained with Dulbecco's modified Eagle's medium (DMEM), supplemented with 10% FBS, 100 U/mL penicillin, and 100 μg/mL streptomycin. AML12 cells were maintained in DMEM/F12 (1:1) (Gibco, 11320-033, Co Dublin, Ireland) medium containing 10% FBS, 1% antibiotics, 1% ITSX (insulin, transferrin, selenium X) (Gibco, 51500-056), and 100 nM dexamethasone.

### Glucose production assay

Primary hepatocytes were maintained in low glucose DMEM for 12 h prior to measurement of glucose production. Cells were washed 3 times with PBS and then stimulated with simvastatin (10 μM) for 6 h in phenol red-free, glucose-free DMEM containing 2 mM sodium pyruvate and 20 mM sodium lactate. Glucose concentrations in the medium were measured with a glucose oxidase assay kit (Thermo, Waltham, MA, USA).

### Western blotting and antibodies

Tissues and cells were homogenized in Tissue Protein Extraction Reagent or Mammalian Protein Extraction Reagent (Thermo). Nuclear and cytoplasmic components were isolated using NE-PER nuclear and cytoplasmic extraction kit (Thermo). Homogenates (20 μg of total protein) were separated by SDS-PAGE and transferred to nitrocellulose membranes.

Antibodies were used against the following proteins: Sirt6, Sirt1, Ac-H3K9, FoxO1, p-FoxO1 (Cell Signaling, Beverly, MA, USA), Ac-lysine, Sirt2, Sirt3 (Abcam, Cambridge, UK), ubiquitin (Santa Cruz Biochemicals, Dallas, TX, USA), Sirt4, lamin B, GAPDH (Bioworld Technology, St Louis Park, MN, USA), Sirt5, Sirt7, Ac-FoxO1 (LifeSpan Biosciences, Seattle, WA, USA), and HSP90 (Enzo Life Sciences, Plymouth Meeting, PA, USA).

### RNA isolation and real-time quantitative RT-PCR (qPCR)

Total RNA was extracted from liver tissue or cell cultures using RNA Iso kit (TaKaRa, Tokyo, Japan). First-strand cDNA was generated using the random hexamer primer provided in the first-strand cDNA synthesis kit (Applied Biosystems, Foster City, CA, USA). Specific primers for each gene were designed using PrimerBank (Table S2). qPCR reactions were performed in 10 μl containing 10 ng of reverse-transcribed total RNA, 200 nM of forward and reverse primers, and PCR master mixture in 384-well plates using an ABI Quant6 Flex Real-Time PCR System (Applied Biosystems).

### miRNA and bioinformatic analyses

For miRNA qPCR, cDNA was generated from 1 μg of total RNA per sample, using miScript reverse transcription kit (QIAGEN GmbH, Hilden, Germany). qPCR analysis was performed with miScript SYBR Green PCR Kit (QIAGEN GmbH). Values were calculated using the comparative DDCt method and normalized to U6 small RNA. MiR-495 mimic and miR-495 antisense oligonucleotide (ASO) were transfected into AML12 cells using Lipofectamine RNAiMAX Reagent (Invitrogen, Carlsbad, CA, USA). Twenty four hours after transfection, cells were harvested for later use. MiRNAs measured in this work are shown in Table S3.

For screening the potential miRNAs to target Sirt6, the database (www.microrna.org) was analyzed. Among putative miRNAs to bind to mouse Sirt6 3′-untranslated region (UTR), top 10 miRNAs were selected according to the prediction score. After examining all the selected miRNAs expression in the primary hepatocytes treated with simvastatin, rosuvastatin, or atorvastatin, commonly altered- and species conserved-miRNA was finally identified.

For the pathway analysis, among predicted targets of miR-495 (www.microrna.org), the genes with the criteria (i.e., mirSVR scores below -0.5; 2174 genes) were clustered by KEGG pathway using the Database for Annotation, Visualization and Integrated Discovery (DAVID) 6.8 bioinformatics resource (https://david.ncifcrf.gov/).

### Reporter assays

The miRNA 3'-UTR target clone containing mouse Sirt6 3'-UTR (Product ID: MmiT117969) was provided from GeneCopoeia (Rockville, MD, USA). HEK293 cells were co-transfected with the reporter construct and miR-495 mimic or ASO as an inhibitor or its respective control. Forkhead responsive element luciferase (FHRE-Luc) was purchased from Addgene (Watertown, MA, USA). For the FoxO1-promoter reporter gene assay, mouse FHRE-Luc construct (1 μg) was transfected into AML12 cells, together with FoxO1 expression plasmid or empty vector for 24 h, using Lipofectamine 3000 (Invitrogen). Luciferase activity was measured using the Dual-Luciferase reporter assay system (Promega, Madison, WI, USA).

### Statistical analysis

Data are expressed as the mean ± standard error of the mean (SEM). Statistical comparisons were performed using one-way analysis of variance followed by Fisher's *post hoc* analysis. The significance of differences between two groups was determined using Student's unpaired *t*-test. A *p*-value of less than 0.05 was considered significant.

## Results

### Statin treatment downregulates Sirt6 expression while increasing hepatic gluconeogenesis

**Figure 1:**
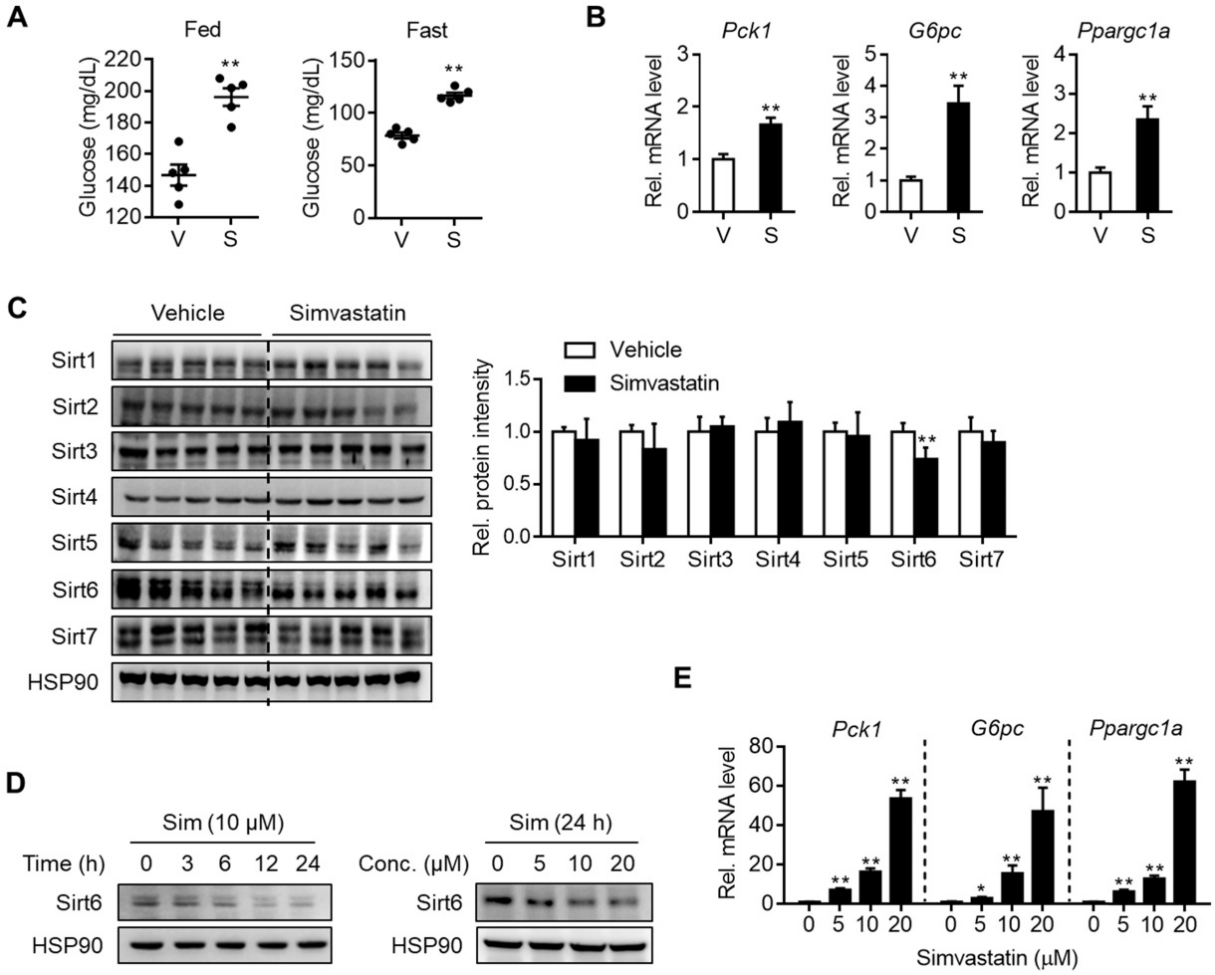
** Downregulation of Sirt6 and increase in hepatic gluconeogenesis by statin.** C57BL/6 mice were administered simvastatin 30 mg/kg i.p. once a day for three consecutive days. **(A)** Blood glucose was measured in fed or fasted mice with simvastatin treatment two or three times, respectively (n = 5). **(B, C)** Two hours after the last simvastatin dosing, liver tissues were harvested and subjected to qPCR and Western blotting analyses (n = 4-5). **(D, E)** Mouse primary hepatocytes were treated with simvastatin (Sim) at indicated concentrations and time points. Western blotting and qPCR analyses were performed. Values are means ± SEM. ^*^, *p <* 0.05 and ^**^, *p <* 0.01 versus vehicle. V, vehicle; S, simvastatin.

To determine whether statin treatment affects normal glycemia, mice were administered with various statins for three consecutive days. Results show that simvastatin increased both fed and fasting blood glucose levels (Figure 1A) without affecting body and liver weights (Figure S1A). The same results were obtained with different statins, i.e., rosuvastatin, atorvastatin, fluvastatin, lovastatin, and pravastatin showed similar effects without significant differences between them (Figures S1A and S1B). Concurrently with this, mRNA levels of gluconeogenesis genes such as *Pck1*, *G6pc,* and *Ppargc1a* were increased in the statin-treated liver (Figures 1B and S1C). Based on previous findings showing that sirtuins affect hepatic gluconeogenesis 16, 17, 19-22 we inferred that statin could alter the expression of sirtuin members. To test this hypothesis, we selected simvastatin as a representative drug for further study and evaluated its effects on the hepatic expression of sirtuins. When mice were treated with simvastatin for three days, Sirt6 was noticeably suppressed by 30% in the liver, while other sirtuins were not changed (Figure 1C). Simvastatin downregulation of Sirt6 protein was further confirmed in mouse primary hepatocytes, and was observed at 5-20 μM of simvastatin in a concentration-dependent manner (Figure 1D). This was paralleled by increases in mRNA levels of gluconeogenesis genes (Figure 1E), consistent with the results observed in the mouse liver. Treatment of hepatocytes with rosuvastatin or atorvastatin also decreased Sirt6 protein in a similar degree to simvastatin (Figure S1D), providing further confirmation of the direct repression of Sirt6 protein by statins.

### Hyperglycemia and gluconeogenesis induced by statin are mitigated by Sirt6 overexpression

**Figure 2:**
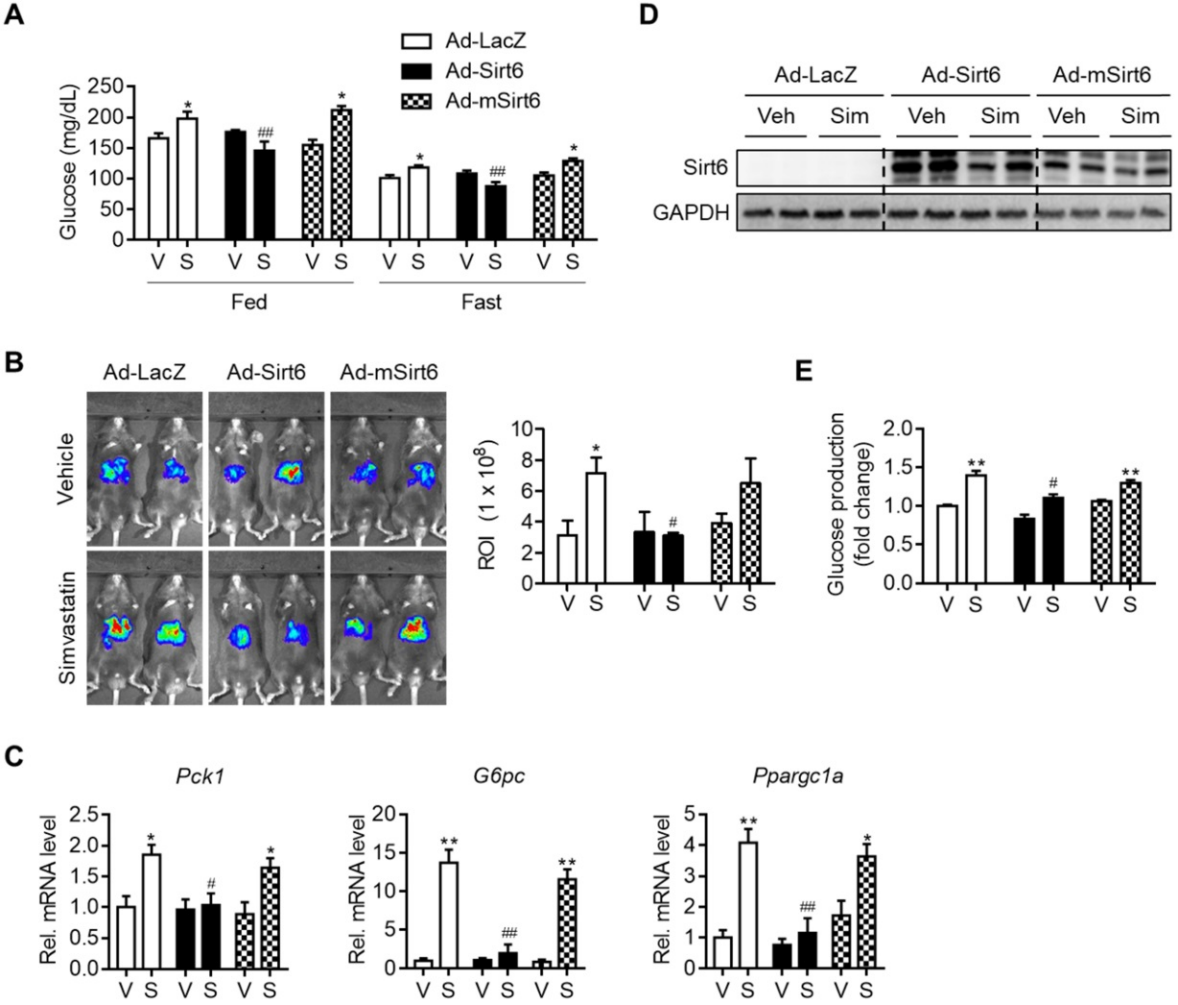
** Attenuation of simvastatin induction of hepatic gluconeogenesis by Sirt6**. C57BL/6 mice were injected intravenously with 1 × 10^9^ pfu of either Ad-LacZ, Ad-Sirt6, or Ad-mSirt6, and then administered simvastatin 30 mg/kg i.p. for 3 days. **(A)** Fed or fast blood glucose levels were measured from mouse tail vein (n = 4-5). **(B)** Hepatic G6Pase-luciferase (Ad-WT G6Pase [-231/+57]-Luc) activities in livers of mice administered simvastatin after 6 h fasting (n = 3-5). **(C, D)** mRNA levels of genes of gluconeogenesis and protein levels of Sirt6 in mice (n = 4-5). **(E)** Primary mouse hepatocytes were infected with either Ad-LacZ, Ad-Sirt6, or Ad-mSirt6 and glucose production assay was performed 6 h after 10 µM simvastatin treatment (n = 3). Values are means ± SEM. ^*^, *p <* 0.05 and ^**^, *p <* 0.01 versus vehicle;^ #^, *p <* 0.05 and ^##^, *p <* 0.01 versus Ad-LacZ. V, vehicle; S, simvastatin.

To establish a causal relationship between changes in gluconeogenesis genes and Sirt6 expression by simvastatin, we performed a Sirt6 overexpression study in mice. Mice were infected with Ad-Sirt6 or Ad-mSirt6 three days prior to statin administration. The higher fed-and fast-blood glucose levels observed with simvastatin were abolished by Sirt6 overexpression (Figure 2A). Conversely, Ad-mSirt6 was without effect. To elaborate upon this finding, we next performed *in vivo* luciferase bioluminescence imaging to measure the transcriptional activity of gluconeogenesis genes in mouse liver, as previously described 23. Mice administered simvastatin exhibited markedly increased G6Pase transcriptional activity *in vivo* compared to control mice (Figure 2B). However, in mice with Ad-Sirt6 but not with Ad-mSirt6, an increase in G6Pase transcriptional activity by simvastatin was not evident. This was corroborated by the qPCR results. Simvastatin-mediated increases in *Pck1*, *G6pc* and *Ppargc1a* were completely abolished by Sirt6 overexpression (Figure 2C). Successful overexpression of Sirt6 in liver with adenovirus was confirmed by Western blotting (Figure 2D). This was further confirmed by glucose production assay and qPCR analysis utilizing mouse primary hepatocytes (Figures 2E and S2). Sirt6 overexpression was also able to prevent hyperglycemia induced by atorvastatin or rosuvastatin in mice (Figure S3). Our results therefore indicate that recovering the expression and deacetylase activity of Sirt6 inhibits statin-mediated hepatic gluconeogenesis and hyperglycemia.

### Statin increases miR-495 as an inhibitor of Sirt6 expression

**Figure 3:**
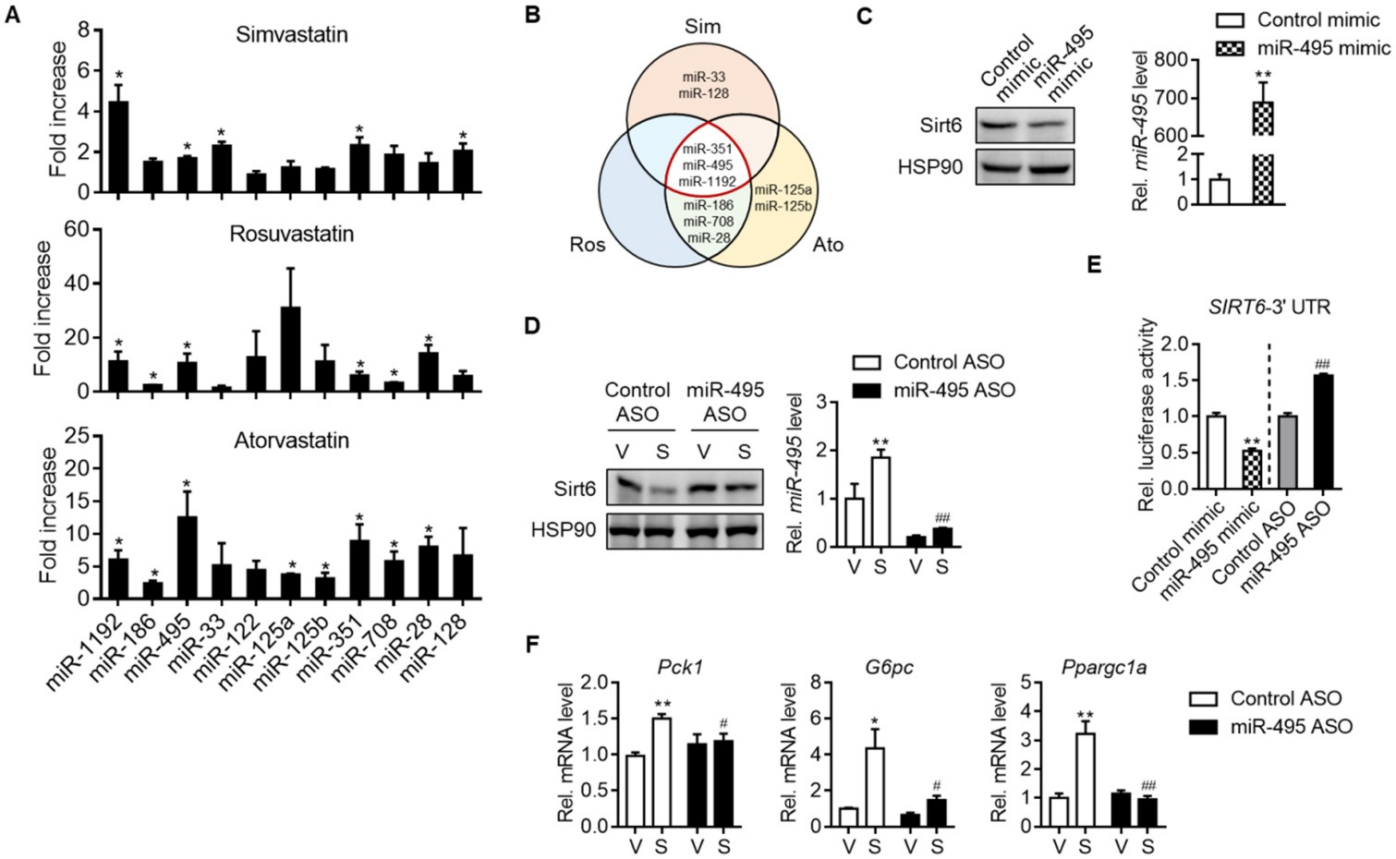
** MiR-495 induction by statin and its role in Sirt6 repression. (A)** Primary hepatocytes were treated with 10 μM simvastatin, rosuvastatin or atorvastatin for 24 h, and the levels of predicted miRNAs targeting Sirt6 were measured (n = 3). **(B)** Venn diagram illustrating miRNAs affected by simvastatin, rosuvastatin, and atorvastatin treatment. **(C)** AML12 cells were transiently transfected with control or miR-495 mimic for 24 h, and levels of Sirt6 protein and miR-495 were examined (n = 6). **(D)** AML12 cells transfected with control or miR-495 antisense oligonucleotide (ASO) were exposed to simvastatin for 6 h (n = 4-5).** (E)** Sirt6 3′-UTR luciferase activities were measured in HEK293 cells transfected with the reporter construct and miR-495 mimic or ASO for 24 h. **(F)** Gluconeogenesis gene transcript levels were measured in AML12 cells treated as in panel (D) (n = 4-5). Values are means ± SEM. ^*^, *p <* 0.05 and ^**^, *p <* 0.01 versus vehicle or control; ^#^, *p <* 0.05 and ^##^, *p <* 0.01 versus control ASO.

We next set out to investigate how statins suppressed Sirt6 protein levels. Sirt6 expression has been studied to be regulated at both transcriptional and post-transcriptional levels. We found that simvastatin increased the mRNA level of Sirt6 in mouse liver and primary hepatocytes (Figures S4A and S4B). Cycloheximide chase analysis showed that simvastatin had no effect on the protein stability of Sirt6 (data not shown). We thus focused on post-transcriptional regulation of Sirt6 by miRNAs. Among the top 10 miRNAs predicted to bind to the 3′-UTR of Sirt6 mRNA, the levels of miR-351, miR-495 and miR-1192 were commonly elevated by simvastatin, rosuvastatin and atorvastatin (Figures 3A and 3B). Since miR-495 is conserved in both humans and mice, we focused on this miRNA in the subsequent experiments.

Next, we determined the effects of miRNA modulations on Sirt6 expression. Whereas transfection of AML12 cells with miR-495 mimic decreased Sirt6 expression (Figure 3C), miR-495 ASO transfection had the opposite effect (Figure 3D). Moreover, the ability of simvastatin to decrease Sirt6 protein was blocked by miR-495 ASO (Figure 3D). No change was confirmed in Sirt6 mRNA by miR-495 mimic or ASO (Figures S4C and S4D), supporting the post-transcriptional inhibition of Sirt6 by miR-495. The direct inhibitory effect of miR-495 on Sirt6 translation was further supported by Sirt6 3′-UTR reporter assay (Figure 3E). Finally, to explore the role of miR-495 in statin-induced hyperglycemia, we examined gluconeogenesis gene expressions after treatment with miR-495 ASO. Silencing of miR-495 with ASO significantly suppressed simvastatin-induced expression of gluconeogenesis genes (Figure 3F). The role of simvastatin in miR-495 induction, Sirt6 protein repression, and mRNA increases in gluconeogenesis genes was also confirmed in human hepatocarcinoma cell line HepG2 (Figures S5A-S5C).

We next asked how statin up-regulates miR-495 expression and wanted to test the possible involvement of mevalonate pathway 24, 25. The ability of simvastatin to induce miR-495 expression and the consequent dysregulation of Sirt6-FoxO1-gluconeogenesis axis were significantly blocked by supplementation of GGPP, which is the intermediate metabolite produced by HMG-CoA reductase, but neither by FPP, cholesterol, nor 25-hydroxycholesterol (Figures S6A-S6C). These results support the concept that inhibition of HMG-CoA reductase and the consequent suppression of GGPP biosynthesis by statin, but not diminished cholesterol, leads to miR-495 up-regulation. In addition, the ability of simvastatin to increase the expression of primary miR-495 was also demonstrated (Figure S6D), indicating the transcriptional induction of miR-495 by statin.

Taken together, these results indicate that Sirt6 is a novel target of miR-495, and that statin-mediated miR-495 induction was responsible for Sirt6 protein repression and concomitant gluconeogenesis gene induction.

### FoxO1 up-regulation as a result of miR-495/Sirt6 dysregulation by statin

**Figure 4:**
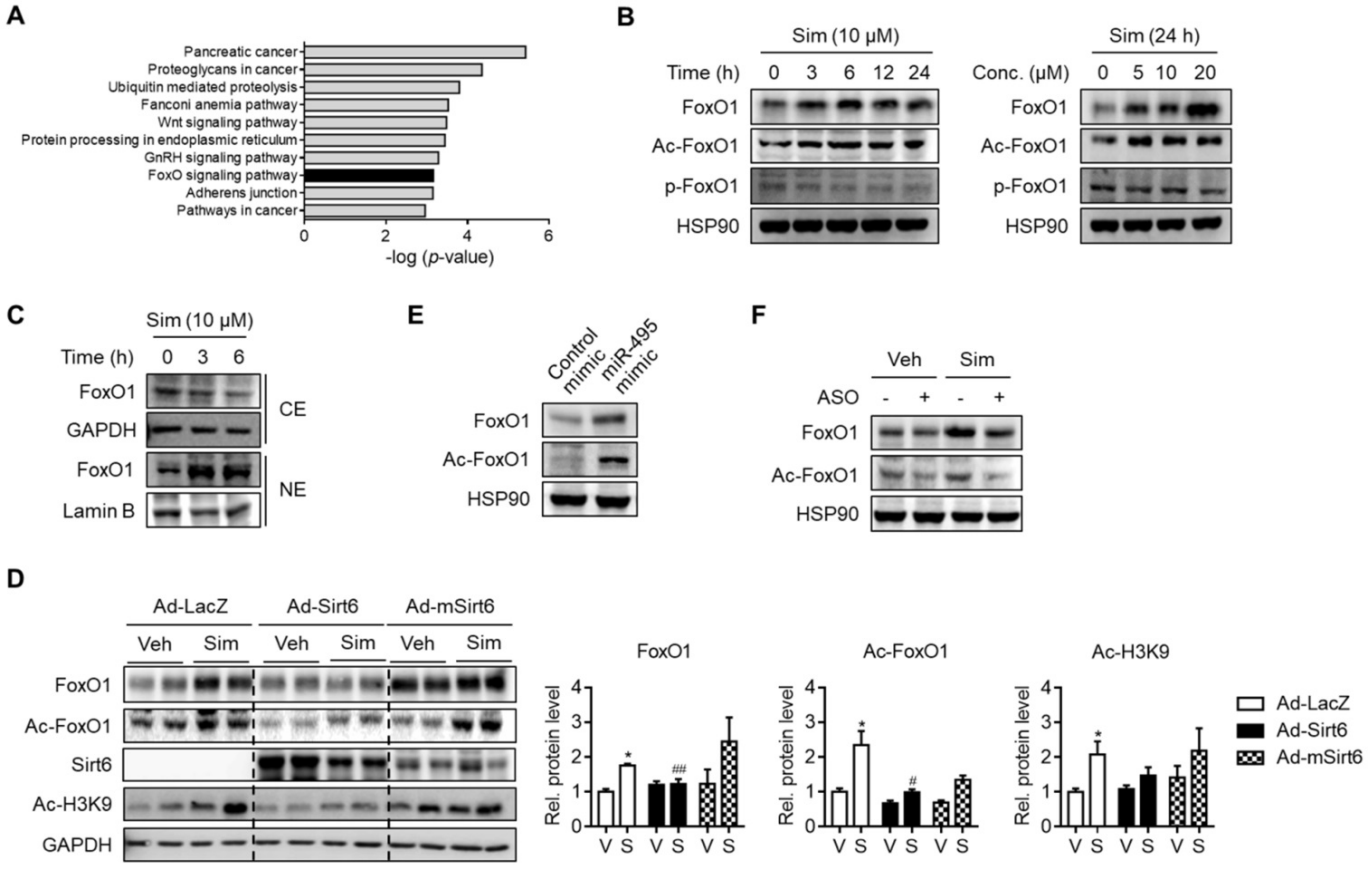
** Up-regulation of FoxO1 by simvastatin. (A)** KEGG pathway analysis targeted by miR-495. The statistical significance of KEGG pathway was ranked according to -log (p-value).** (B, C)** Mouse primary hepatocytes were incubated with simvastatin (Sim) as indicated and protein levels of FoxO1 were determined in whole lysates (B) and in cytosolic (CE) and nuclear extracts (NE) (C). **(D)** Effect of Sirt6 overexpression on total and acetylated FoxO1 expression in liver tissues. H3K9 acetylation (Ac-H3K9) was measured as an indicant for Sirt6 deacetylase activity. **(E)** Effect of miR-495 mimic transfection on total and acetylated FoxO1 expression in AML12 cells. **(F)** Effect of miR-495 ASO transfection on FoxO1 level after incubation with simvastatin for 6 h. Values are means ± SEM. ^*^, *p <* 0.05 versus vehicle; ^#^, *p <* 0.05 and ^##^, *p <* 0.01 versus Ad-LacZ. V, vehicle; S, simvastatin.

In order to locate the downstream targets of statin-mediated Sirt6 repression, we further analyzed the predicted target genes of miR-495. KEGG pathway analysis suggested that the genes involved in the FoxO signaling pathway were significantly enriched (Figure 4A). Accordingly, we hypothesized that FoxO1, a major FoxO member for hepatic gluconeogenesis, is associated with Sirt6 regulation of statin-induced glucose production. In support of this notion, FoxO1 has been found both by ourselves and in other studies to be deacetylated by Sirt6 17, 26. Results in primary hepatocytes showed that FoxO1 protein expression and its acetylation were increased by simvastatin in a time- and concentration-dependent manner (Figure 4B). Although phosphorylation of FoxO1 was not affected by simvastatin (Figure 4B), its nuclear level was markedly increased, whereas its cytosolic level was decreased (Figure 4C), suggesting that FoxO1 acetylation determines subcellular localization. Because simvastatin up-regulation of FoxO1 was so pronounced, we tested the hypothesis of simvastatin control of the Sirt6-FoxO1 axis by adenoviral overexpression of Sirt6 in mice. Sirt6 overexpression in mice abolished the increases in total- and acetylated-FoxO1 levels by simvastatin (Figure 4D).

We further examined the effect of miR-495 on the regulation of FoxO1. Transfection with miR-495 mimic - a condition identical to Sirt6 repression by statin - markedly raised the levels of total- and acetylated-FoxO1 (Figure 4E). Correspondingly, simvastatin-induced FoxO1 induction was diminished by miR-495 ASO (Figure 4F). Taken together, these findings suggest that statin up-regulates FoxO1 by miR-495-mediated Sirt6 inhibition.

**Figure 5:**
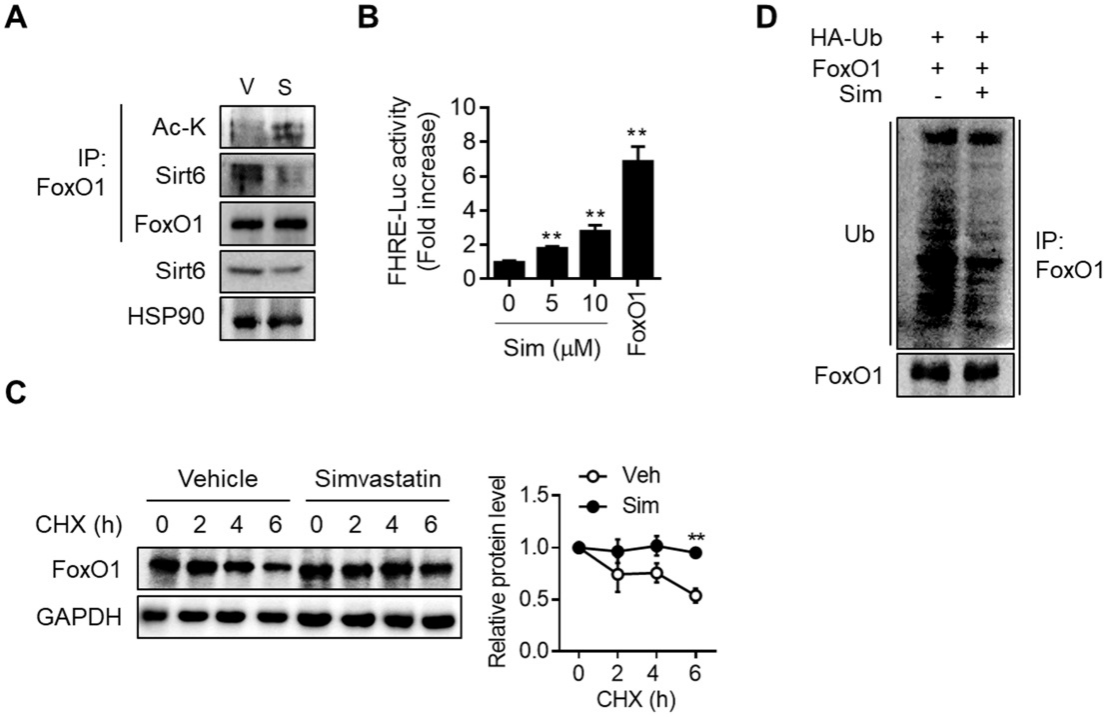
** Effect of simvastatin on protein stability of FoxO1. (A)** Mouse primary hepatocytes were treated with 10 µM simvastatin for 6 h and Co-IP was performed.** (B)** AML12 cells were transfected with FHRE-Luc promoter and treated with simvastatin (Sim). FoxO1-expressing construct was used as a reference (n = 8). **(C)** Mouse primary hepatocytes pretreated with vehicle or simvastatin for 30 min were treated with cycloheximide (CHX, 25 µg/mL) for indicated times, and FoxO1 protein levels were compared (n = 3).** (D)** AML12 cells transfected with FoxO1- and Ub-expressing plasmids were incubated with vehicle or simvastatin for 6 h in the presence of 1 µM MG132. Cell lysates were immunoprecipitated with anti-FoxO1 antibody and immunoblotted with anti-ubiquitin antibody. Values are means ± SEM. ^**^, *p <* 0.01 versus vehicle. V, vehicle; S, simvastatin.

Direct interaction between Sirt6 and FoxO1 and the consequent increase in FoxO1 promoter activity upon simvastatin treatment, were demonstrated by co-immunoprecipitation (co-IP) and FoxO1 promoter-luciferase assay, respectively (Figures 5A and 5B). Upon cytosolic localization, FoxO1 is quickly degraded and inactivated by the proteasome-ubiquitination pathway. Consistent with the simvastatin-induced increase in the nuclear level of FoxO1, cycloheximide chase analysis demonstrated that protein stability in FoxO1 was markedly increased by simvastatin (Figure 5C). Ubiquitination of FoxO1 was also found to be inhibited by simvastatin (Figure 5D). Sirt6 repression and the parallel increase in FoxO1 expression was also demonstrated in the liver of mice treated with atorvastatin and rosuvastatin (Figure S7A). Likewise, treatment of hepatocytes with these statins increased both acetylated- and nuclear-FoxO1 levels as well as FoxO1 promoter luciferase activity, changes that were prevented by Sirt6 overexpression (Figures S7B-S7E).

### Amelioration of hyperglycemia by combinatory treatment of Sirt6 activator with statin

**Figure 6:**
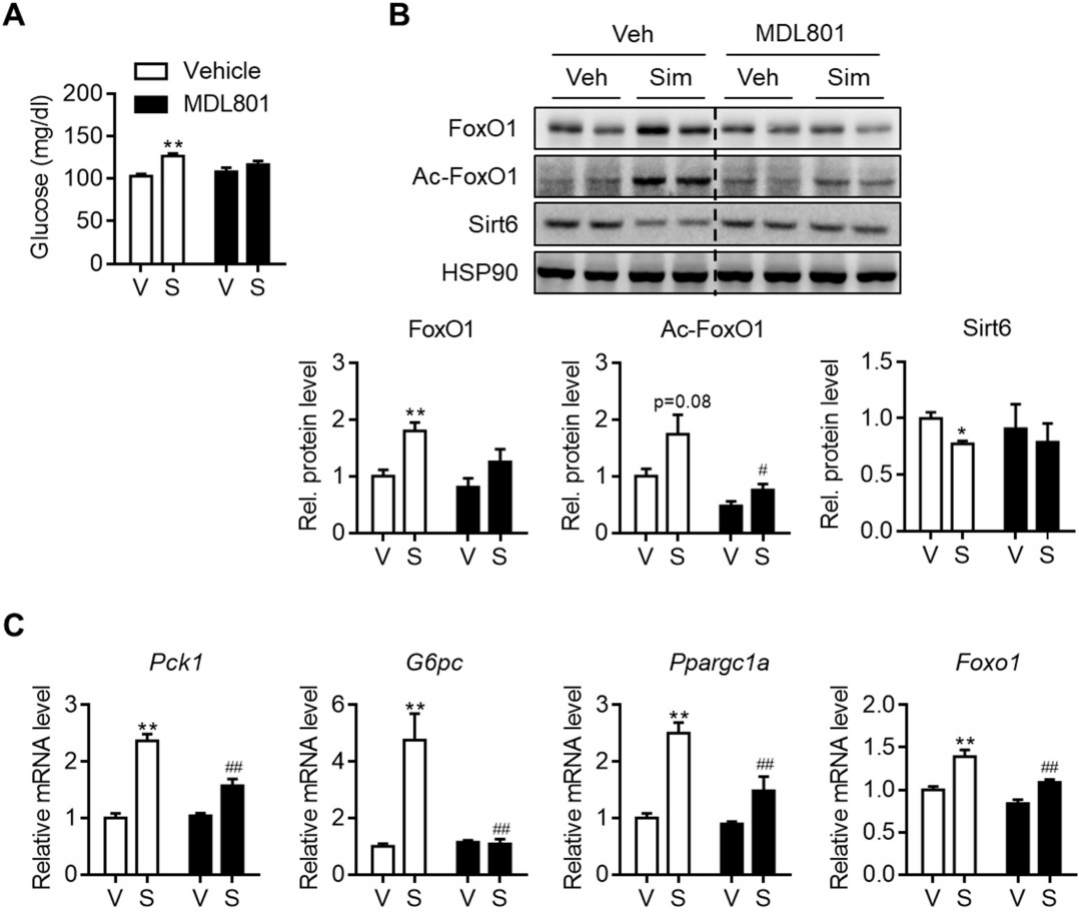
** Effect of Sirt6 activator on simvastatin induced hyperglycemia.** C57BL/6 mice were administered MDL801 (100 mg/kg) orally in combination with simvastatin intraperitoneally for three days.** (A)** Fast blood glucose was measured (n = 4-5). **(B)** FoxO1 and Sirt6 expression in mouse liver after MDL801 and simvastatin treatment. **(C)** mRNA levels of gluconeogenesis genes were measured in mice as described in (B). Values are means ± SEM.^ *^, *p <* 0.05 and ^**^, *p <* 0.01 versus vehicle; ^#^, *p <* 0.05 and^ ##^, *p <* 0.01 versus simvastatin alone. V, vehicle; S, simvastatin.

Allosteric activators of Sirt6 deacetylase have been reported 27, 28. Because Sirt6 down-regulation by statins was shown to be a cause of hyperglycemia, we further investigated whether Sirt6 activation by chemical activators, specifically MDL801 and fucoidan, could prevent this. To test this hypothesis, C57BL/6 mice were administered with MDL801 for three days, in combination with simvastatin. Increases in blood glucose were observed in mice administered simvastatin alone, but not in mice co-treated with MDL801 (Figure 6A). While Sirt6 repression and FoxO1 up-regulation were observed in livers of simvastatin-treated mice, these were completely abolished by co-treatment with MDL801 (Figure 6B). Accordingly, increases in the hepatic mRNA levels of *Pck1*, *G6pc,* and *Ppargc1a* by simvastatin were reversed by combined treatment with MDL801 (Figure 6C). Administering fucoidan to mice was similarly observed to prevent statin-induced hyperglycemia and gluconeogenesis induction (Figures S8A and S8B).

### Dysregulation of miR-495/Sirt6 and gluconeogenesis genes in patients taking statins

**Figure 7:**
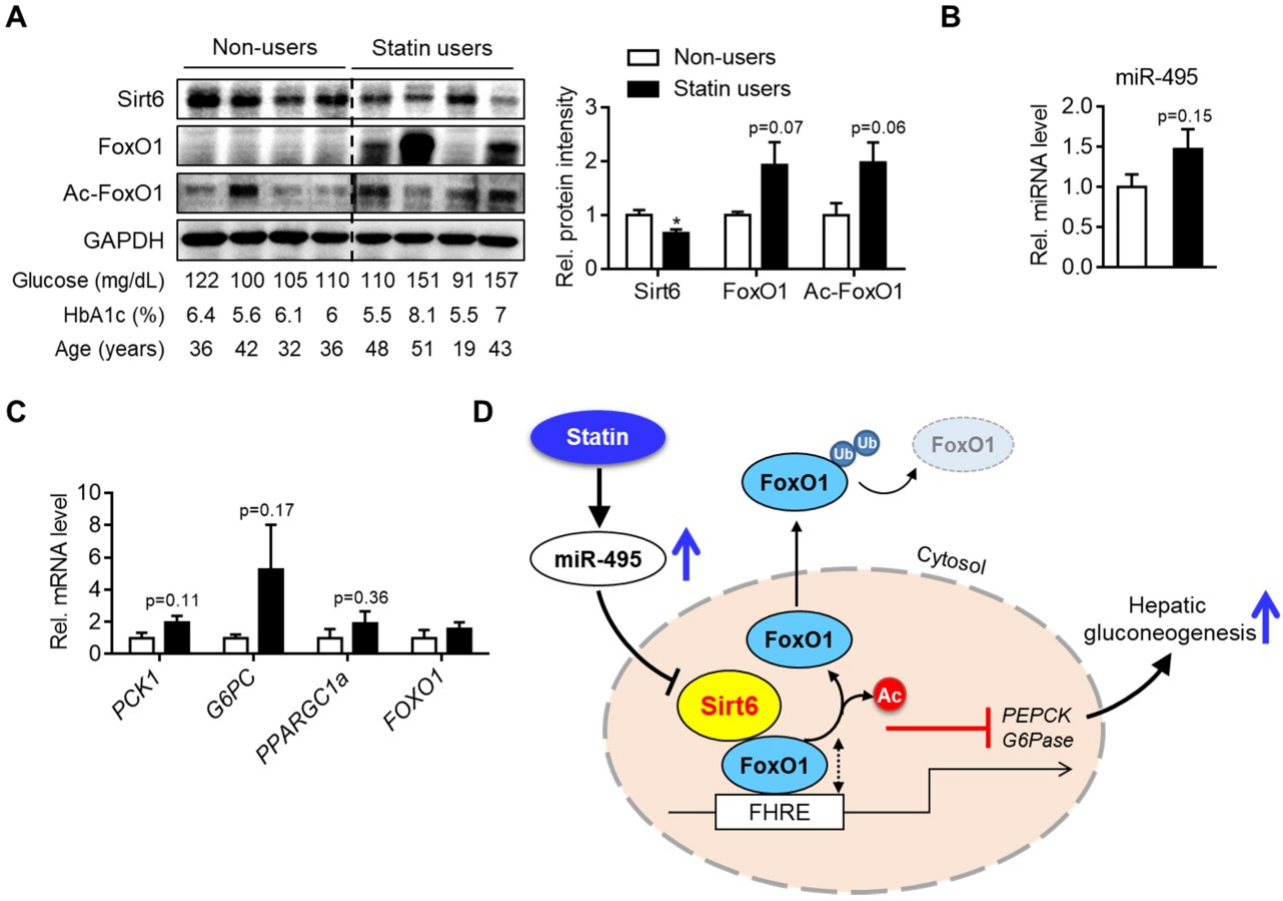
** Hepatic Sirt6 expression in statin users. (A)** Hepatic expression of Sirt6 and total- and acetylated-FoxO1 were compared between statin users and non-users by Western blotting. **(B, C)** mRNA levels of miR-495 and gluconeogenesis genes in human liver tissues (n = 4). **(D)** Proposed summary. Values are means ± SEM. ^*^, *p <* 0.05 versus non-users.

To demonstrate the clinico-pathological influences of Sirt6 in the livers of patients taking statins, we finally analyzed the level of miR-495/Sirt6 and its downstream effectors in the human liver. As shown in Figure 7A, Sirt6 expression was significantly decreased in livers of statin users compared to those of non-users. Conversely, FoxO1 expression and its acetylation tended to increase in the statin group. MiR-495 levels were also moderately increased in livers of statin users (Figure 7B). qPCR analysis confirmed the up-regulation of *PCK1* and *G6PC* without statistical significance (Figure 7C). Overall, we established that statin-induced miR-495 suppresses the Sirt6 protein level in hepatocytes, which leads to up-regulation of the FoxO1 transcription factor and, consequently, enhancement of hepatic gluconeogenesis (Figure 7D).

## Discussion

The results of the present study confirmed previous reports of increased gluconeogenesis caused by statins in hepatocytes 9, 10, and provided further evidence that an increase in blood glucose was observed even with acute treatment of statins in mice. This study has demonstrated, firstly, that statin treatment suppressed hepatic expression of Sirt6 in humans as well as mice. This is an important discovery, since genetic overexpression of Sirt6 or co-administration of Sirt6 activators almost completely prevented increases in hepatic gluconeogenesis gene expression and the concomitant elevation in blood glucose levels following statin treatment. We have thus identified the molecular mechanism mediated by statins at the levels of upstream and downstream of Sirt6. We have discovered that statin increases miR-495 expression, which causes Sirt6 repression, which in turn promotes acetylation and stabilization of FoxO1, resulting in increased hepatic gluconeogenesis. Our study thus identifies the molecular basis of dysglycemia due to statin use, as well as a preventative measure for this.

Several pathways have been identified as a potential mechanism involved in the effect of statin on insulin resistance 29. Statin impairs the insulin-secreting ability of pancreatic beta cells 25, 30, 31, decreases insulin signaling in skeletal muscle 32, and exacerbates adipose inflammation while suppressing white fat browning 24, 33, 34. Another potential mechanism of statin-mediated insulin resistance that has been suggested is an increase in hepatic gluconeogenesis. In hepatocytes, statin induces autophagy and activates pregnane X receptor to induce gluconeogenesis genes 9, 10. Given that local concentration of statin in the liver is doubled, whereas in other tissues it is only one third as high compared to that of serum 35, it is conceivable that hepatic glucose production is a primary determinant of the dysglycemia associated with statin treatment.

Hepatic gluconeogenesis induced by statin varies depending on the experimental conditions. For example, whereas Wang et al., have reported the hyperglycemic effects of chronic statin treatment (16 weeks) in mice fed a high-fat diet 10, Gotoh and Negishi have posited a decrease in gluconeogenesis genes by acute simvastatin treatment (50 mg/kg, orally once daily for two days) in mice 9. In this study, consistent with the study of Wang et al., we observed hyperglycemia as a result of enhanced induction of gluconeogenesis genes by statin. Interestingly, our results indicate that there was no differential effect on hepatic gluconeogenesis between different varieties of statins. These accords with the findings of previous meta-analysis reports that although the statin-induced hyperglycemia effect is dose-dependent, it is not a specific statin feature 8.

The present study presents evidence that statins promote activation of the FoxO1 transcription factor and that this was mediated by a decrease in Sirt6. Simvastatin treatment increased expression of FoxO1 and Ac-FoxO1 in primary hepatocytes and in mice, which was completely abolished by Sirt6 overexpression, indicating that Sirt6 suppression was the cause of the FoxO1 up-regulation induced by statins. Regulation of FoxO transcription factors occurs through reversible phosphorylation and acetylation. FoxO1 is phosphorylated via the insulin signaling cascade, resulting in its cytoplasmic retention and proteasomal degradation. Acetylation of FoxO1 is regulated by sirtuins and acetyltransferases, although its consequences are controversial. FoxO1 deacetylation by Sirt1 or Sirt6 respectively increased and decreased its transcriptional activity 17, 36, while its acetylation by coactivator p300 has been shown to stimulate FoxO1 induced transcription 36, 37. In full accordance with previous findings on the Sirt6-FoxO1 axis 17, nuclear export and the consequent proteasomal degradation of FoxO1 were inhibited by statins. As a result, FoxO1-dependent promoter activity was increased by statin. Our current results show the first, unique mode of FoxO1 regulation triggered by statins, namely an increase in its acetylation and protein stability, without any corresponding effect on its phosphorylation, thus enhancing hepatic gluconeogenesis.

Another important finding of our study is the discovery of miR-495 as an inhibitor of Sirt6 and its role in statin-induced gluconeogenesis. Contrary to down-regulation of Sirt6 protein upon statin treatment, mRNA expression of Sirt6 was markedly increased, raising the possibility of a potential modification at the post-transcription level. We further found that miR-495 induction by statin occurred at a transcriptional level and that miR-495-Sirt6-FoxO1-gluconeogenesis pathway affected by statin was the result of suppression of mevalonate pathway byproduct GGPP but not of cholesterol biosynthesis. MiRNAs heavily impact gene expression, thus coordinating metabolism, and previous studies of statin have shown that it modifies the expression of numerous miRNAs. We have newly identified miR-495 here as a negative regulator of Sirt6, both in mice and the human liver. Many of the statins we tested shared the ability to up-regulate miR-495 expression, deteriorating the expression and function of Sirt6 in inhibiting the FoxO1 transcription factor and leading to increased gluconeogenesis. Our causality study showed that miR-495 mimic transfection had the same effect as statin treatment on Sirt6 repression, FoxO1 acetylation, and gluconeogenesis gene induction. Correspondingly, the ability of statin to increase gluconeogenesis gene induction was blocked by miR-495 inhibition. MiR-495 has been found to be involved in regulating cell proliferation, apoptosis, and inflammation 38, but its potential role in liver biology and energy metabolism has proved elusive. In the present study, we have shown the novel role of miR-495 in regulating Sirt6 expression and hepatic glucose metabolism.

Our study is the first to report the dysregulation by statins of the Sirt6-FoxO1 pathway in the human liver. Western blotting analysis with human liver obtained from patients who had taken statins demonstrated significant repression of Sirt6 compared to the non-users. Expression of FoxO1 and its acetylation level tended to increase without statistical significance in the statin group. It is worth mentioning that one in four of the patients taking statin whose blood glucose was 151 mg/dL and HbA1c was 8.1% had exhibited exceedingly high level of FoxO1 (Figure 7A). However, the scarcity of human liver samples from statin users is a limitation of the present study.

In summary, we report that statin treatment i) acutely increases hepatic gluconeogenesis and blood glucose levels in mice; ii) represses Sirt6 protein expression, thereby increasing FoxO1 transactivation and gluconeogenesis genes; 3) increases miR-495, which mediates downregulation of Sirt6 protein; and finally, 4) that the hyperglycemia induced by statin was preventable by co-treatment with a Sirt6 activator. Therefore, maintaining the level or activity of Sirt6 is likely to be effective in preventing statin-induced risk of diabetes.

## Supplementary Material

Supplementary figures and tables.

## Abbreviations

Sirt6: sirtuin 6
FoxO1: forkhead box protein O1
PGC1α: peroxisome proliferator-activated receptor gamma coactivator 1-alpha
PEPCK: phosphoenolpyruvate carboxykinase
G6Pase: glucose-6-phosphatase
FHRE: forkhead responsive element
ASO: antisense oligonucleotide
