# Supplementary material for: Statin suppresses sirtuin 6 through miR-495, increasing FoxO1-dependent hepatic gluconeogenesis
Source: Theranostics. 2020 Sep 15;10(25):11416–27. doi: 10.7150/thno.49770 (PMC7545997; doi:10.7150/thno.49770)
Supplement: Supplementary file 1 — Supplementary figures and tables. [file thnov10p11416s1.pdf]

Statin suppresses sirtuin 6 through miR-495, increasing FoxO1-dependent hepatic gluconeogenesis

Min Yan Shi[1,*], In Hyuk Bang[1,*], Chang Yeob Han[2,*], Dae Ho Lee[3], Byung-Hyun Park[1,$] and Eun Ju Bae[2,$]

[1]Department of Biochemistry and Molecular Biology, Chonbuk National University Medical School, Jeonju, Jeonbuk 54896, Republic of Korea
[2]College of Pharmacy, Chonbuk National University, Jeonju, Jeonbuk 54896, Republic of Korea
[3]Department of Internal Medicine, Gil Medical Center, Gachon University College of Medicine, Incheon 21565, Republic of Korea

[*] Equal contribution

[$] Corresponding authors

Correspondence to: Byung-Hyun Park; email: bhpark@jbnu.ac.kr; phone: +82-63-270-3139

Correspondence to: Eun Ju Bae; email: ejbae7@jbnu.ac.kr; phone: +82-63-219-5650

Contents

1. Supplementary tables

2. Supplementary figures

1. Supplementary tables

Table S1. The characteristics of study subjects

Case	Age	Sex	BMI (kg/m^2)	Statin (mg/day)	Glucose (mg/dL)	HbA1c (%)	Insulin (μU/mL)	Total cholesterol (mg/dL)	TG (mg/dL)	HDL-C (mg/dL)	HOMA -IR
1	36	F	40.6	No	122	6.4	32.9	224	153	61	9.9
2	42	F	40.8	No	100	5.6	11.2	238	291	44	2.8
3	32	F	40.7	No	105	6.1	20.3	294	260	44	5.3
4	36	F	41.3	No	110	6	31.7	261	158	40	8.6
5	48	M	36.4	Pitavastatin (2 mg)	110	5.5	12.17	193	376	44	3.3
6	51	M	34.8	Atorvastatin (10 mg)	151	8.1	17.5	164	114	44	6.5
7	19	F	36.6	Atorvastatin (10 mg)	91	5.5	15.8	190	124	59	3.5
8	43	F	40.8	Rosuvastatin (10 mg)	157	7	12.72	223	236	43	4.9

BMI, body mass index; HbA1c, hemoglobin A1c; TG, triglycerides; HDL-C, high-density lipoprotein cholesterol; HOMA-IR, homeostasis model assessment-insulin resistance

Table S2. Sequences and accession numbers for primers (forward, FOR; reverse, REV) used in qPCR

Gene	Sequences for primers	Accession No.
Actb	FOR: ATGGAGGGGAATACAGCC<br>REV: TTCTTTGCAGCTCCTTCGTT	NM_007393
Foxo1	FOR: GAGTGGATGGTGAAGAGCGT<br>REV: TGCTGTGAAGGGACAGATTG	NM_019739
Sirt6	FOR: GGCTACGTGGATGAGGTGAT<br>REV: GGCTTGGGCTTATAGGAACC	NM_181586
Pck1	FOR: TGTCTTCACTGAGGTGCCAG<br>REV: CTGGATGAAGTTTGATGCCC	NM_011044
G6pc	FOR: ACACCGACTACTACAGCAACA<br>REV: CCTCGAAAGATAGCAAGAGTA	NM_008061
Ppargc1a	FOR: GGATTGAAGTGGTGTAGCGAC<br>REV: GCTCATTGTTGTACTGGTTGGA	NM_008904
ACTB	FOR: CATGTACGTTGCTATCCAGGC<br>REV: CTCCTTAATGTCACGCACGAT	NM_001101
FOXO1	FOR: TCGTCATAATCTGTCCCTACACA<br>REV: CGGCTTCGGCTCTTAGCAAA	NM_002015
SIRT6	FOR: CCCACGGAGTCTGGACCAT<br>REV: CTCTGCCAGTTTGTCCCTG	NM_001193285
PCK1	FOR: GCAAGACGGTTATCGTCACCC<br>REV: GGCATTGAACGCTTTCTCAAAAT	NM_002591
G6PC	FOR: GTGTCCGTGATCGCAGACC<br>REV: GACGAGGTTGAGCCAGTCTC	NM_000151
PPARGC1A	FOR: TCTGAGTCTGTATGGAGTGACAT<br>REV: CCAAGTCGTTCACATCTAGTTCA	NM_013261

Table S3. Sequences for primers used in qPCR for miRNAs

miRNAs	Sequences for primers
RNU6B	AATTCGTGAAGCGTTCCATATT
miR-1192	AAACAAACAAACAGACCAAATT
miR-186	CAAAGAATTCTCCTTTTGGGCT
miR-495	AAACAAACATGGTGCACTTCTT
miR-33	GTGCATTGTAGTTGCATTGCA
miR-122	TGGAGTGTGACAATGGTGTTTG
miR-125a-5p	TCCCTGAGACCCTTTAACCTGTGA
miR-125-b-5p	TCCCTGAGACCCTAACTTGTGA
miR-351	TCCCTGAGGAGCCCTTTGAGCCTG
miR-708	AAGGAGCTTACAATCTAGCTGGG
miR-28	AAGGAGCTCACAGTCTATTGAG
miR-128	TCACAGTGAACCGGTCTCTTT
Primary miR-495 (mouse)	FOR: CCTTCACACTCAGGCACACT<br>REV: GTCCTCCCTCTGGTCCATG

2. Supplementary figures

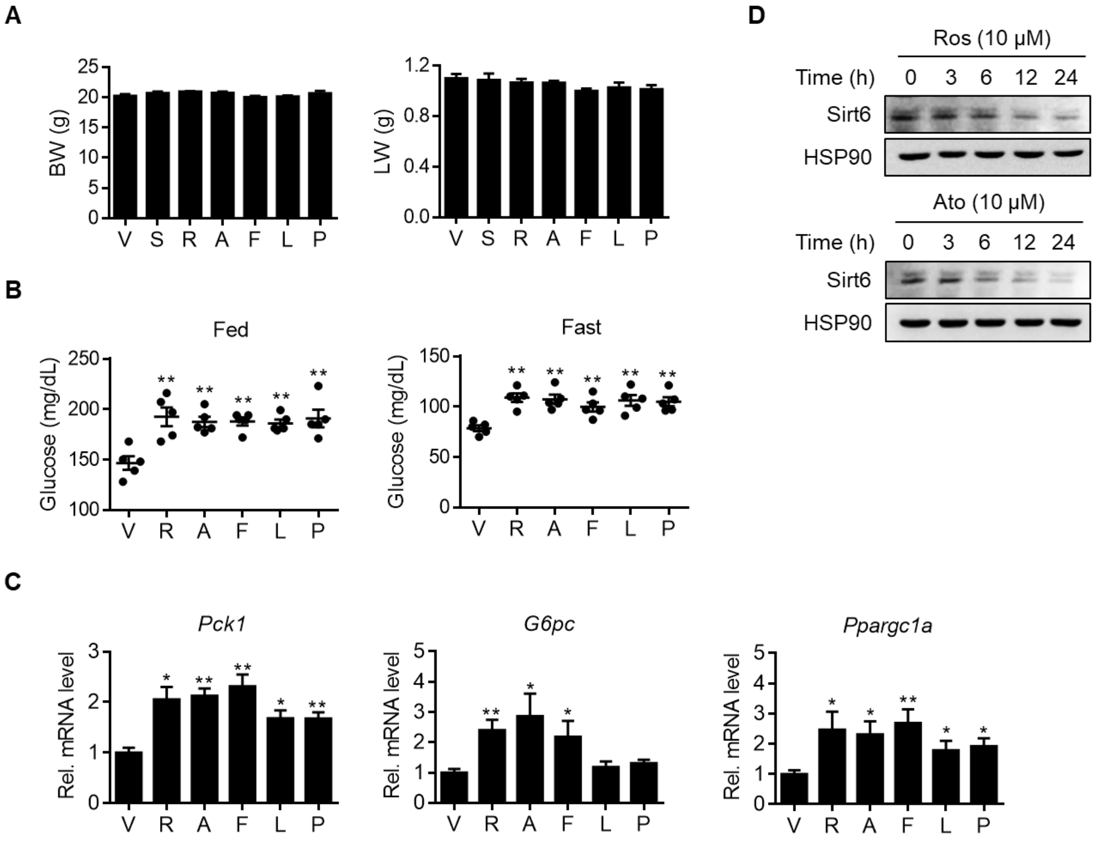

Figure S1. Effects of various statins on glycemia and Sirt6 expression. C57BL/6 mice were administered various statins 30 mg/kg i.p. once a day for three consecutive days. **(A)** Body and liver weights were measured after sacrifice (n = 5). **(B)** Fed or fasted blood glucose were measured (n = 5). **(C)** mRNA levels for gluconeogenesis genes were analyzed by qPCR (n = 4-5). **(D)** Mouse primary hepatocytes were treated with rosuvastatin (Ros) or atorvastatin (Ato) for indicated time points. Sirt6 expression was measured by Western blotting. Values are means ± SEM. *, $p<0.05$ and **, $p<0.01$ versus vehicle. V, vehicle; S, simvastatin; R, rosuvastatin; A, atorvastatin; F, fluvastatin; L, lovastatin; P, pravastatin.

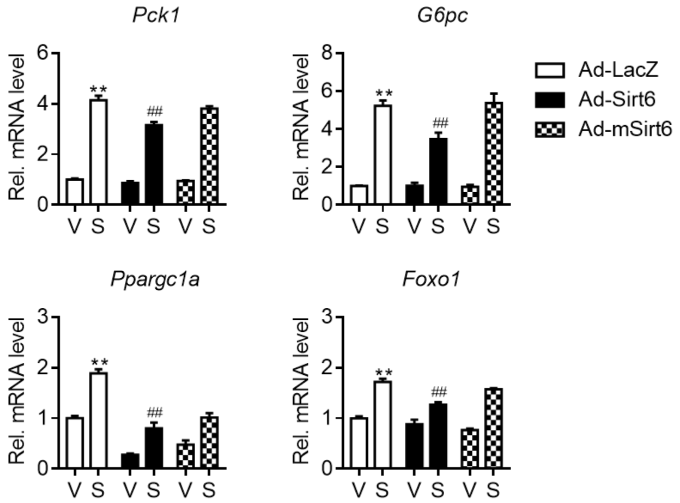

Figure S2. Gluconeogenesis gene expression in primary hepatocytes after simvastatin treatment. Primary mouse hepatocytes were infected with adenoviruses of Sirt6 and incubated with 10 μM simvastatin for 24 h, and RNA was extracted for qPCR analysis (n = 4-6). Values are means ± SEM. **, $p<0.01$ versus vehicle; $^{\#\#}$, $p<0.01$ versus Ad-LacZ. V, vehicle; S, simvastatin.

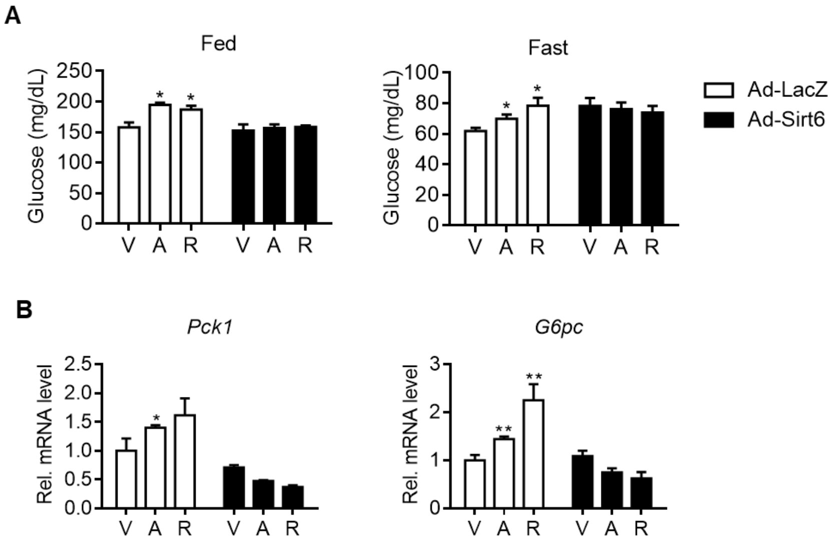

Figure S3. Blood glucose and liver mRNA of gluconeogenesis genes after administration of atorvastatin or rosuvastatin in mice. C57BL/6 mice were administered atorvastatin or rosuvastatin (30 mg/kg) once a day for three consecutive days. **(A)** Fed glucose was measured 9 h after the second dosing of statins, while fast glucose was measured 2 h after the last statin dosing (n = 4-5). **(B)** RNA was extracted from mice liver tissues and used for qPCR analysis (n = 4-5). Values are means ± SEM. *, $p<0.05$ and **, $p<0.01$ versus vehicle. V, vehicle; A, atorvastatin; R, rosuvastatin.

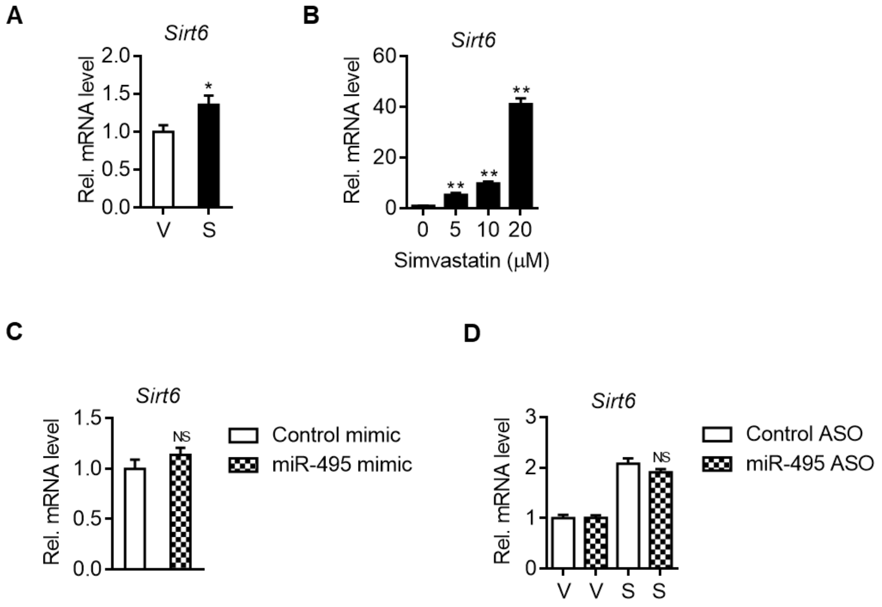

Figure S4. Effect of simvastatin, miR-495 mimic, or miR495 ASO on the expression of Sirt6. (A) Mice were administered simvastatin (30 mg/kg i.p.) once a day for three consecutive days, after which hepatic Sirt6 mRNA levels were examined (n = 5). **(B)** Mouse primary hepatocytes were treated with simvastatin for 24 h, and Sirt6 mRNA was measured (n = 6). **(C, D)** Sirt6 mRNA levels were examined in AML12 cells transfected with control or miR-495 mimic (C) or in cells treated with simvastatin after transfection with control or miR-495 ASO (D). Values are means ± SEM (n = 6). *, $p<0.05$ and **, $p<0.01$ versus vehicle; NS, not significant

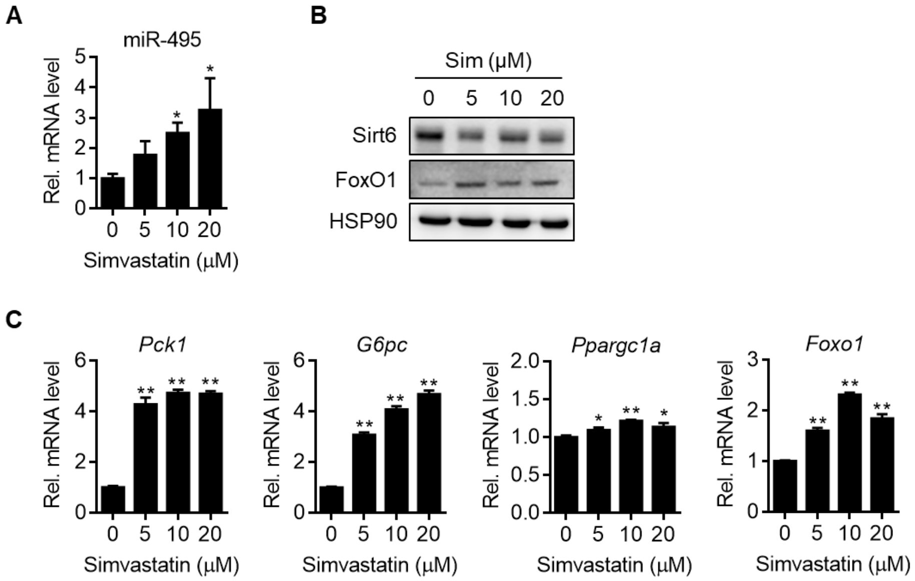

Figure S5. miR-495/Sirt6/FoxO1 regulation by simvastatin in HepG2 cells. HepG2 cells were treated with simvastatin for 24 h, and the level of miR-495 **(A)**, the protein levels of Sirt6 and FoxO1 **(B)**, and the mRNA levels of gluconeogenesis genes **(C)** were analyzed. Values are means ± SEM (n = 5-6). *, $p<0.05$ and **, $p<0.01$ versus vehicle.

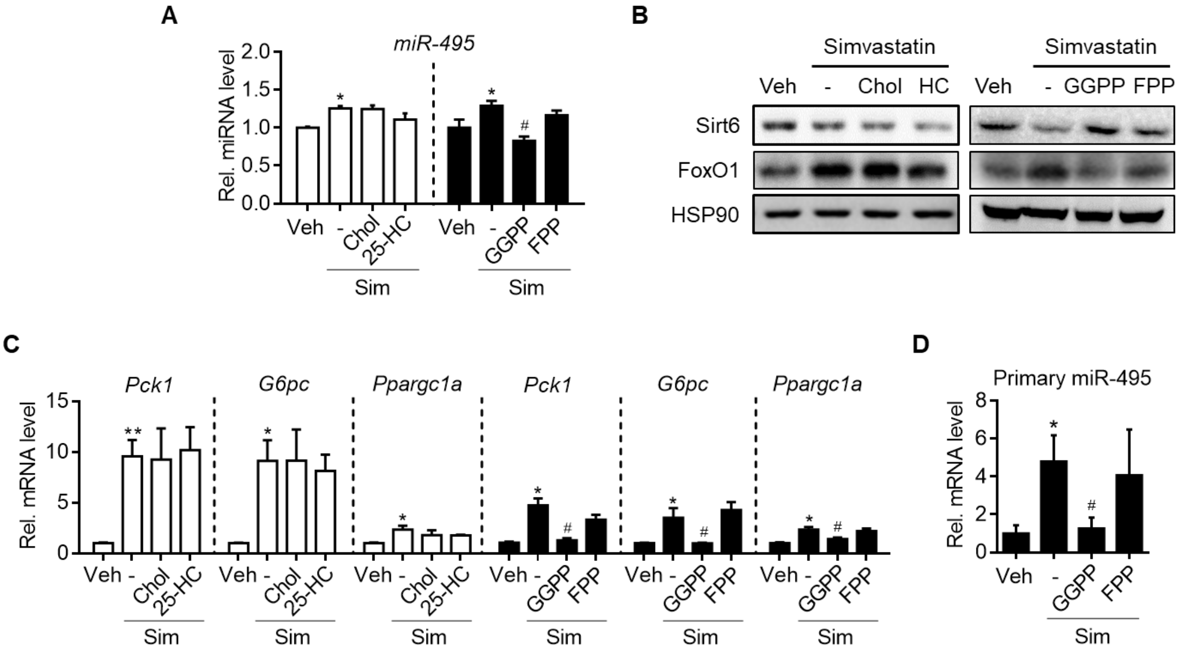

Figure S6. GGPP supplementation restores miR-495/Sirt6/FoxO1/gluconeogenesis pathway affected by simvastatin. Primary hepatocytes were pretreated with 10 μM cholesterol (Chol), 10 μM 25-hydroxycholsterol (25-HC), 10 μM geranylgeranyl pyrophosphate (GGPP), or 5 μM farnesyl pyrophosphate (FPP) prior to 10 μM simvastatin incubation for 24 h, and the level of miR-495 (A), the protein levels of Sirt6 and FoxO1 (B), the mRNA levels of gluconeogenesis genes (C), and the level of primary form of miR-495 were analyzed (D). Values are means ± SEM (n = 4-6). *, p<0.05 and **, p<0.01 versus vehicle.

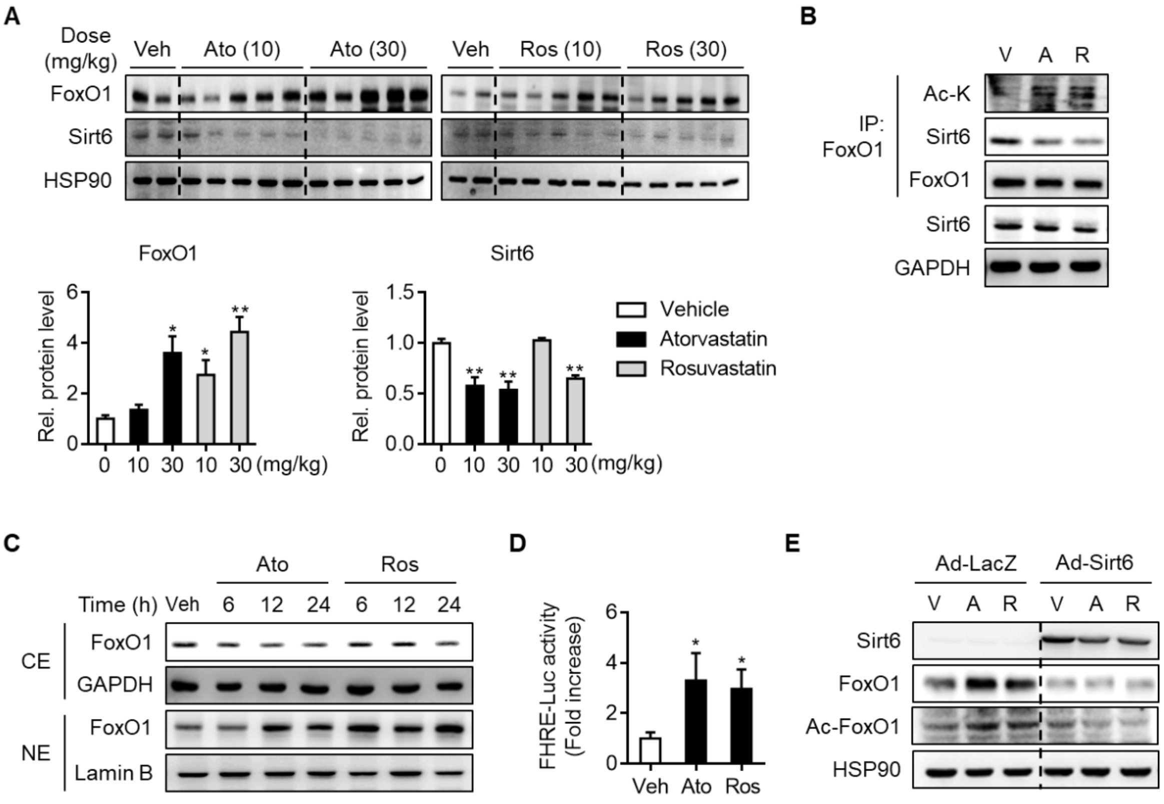

Figure S7. Repression of Sirt6 by various statins. (A) C57BL/6 mice were administered atorvastatin (Ato) or rosuvastatin (Ros) for 3 consecutive days and their liver was analyzed for the expression of Sirt6 and FoxO1 (n = 4-5). **(B)** Mouse primary hepatocytes were treated with10 μM atorvastatin or rosuvastatin and FoxO1 immunoprecipitates were analyzed for Sirt6 and acetylated FoxO1. **(C)** Mouse primary hepatocytes were treated with10 μM atorvastatin or rosuvastatin, while cytosolic (CE) and nuclear (NE) FoxO1 were analyzed by Western blotting. **(D)** AML12 cells were transfected with FHRE-Luc promoter and then treated with atorvastatin or rosuvastatin for 6 h. FHRE luciferase activity was analyzed (n = 5-6). **(E)** Mouse primary hepatocytes were infected with Ad-LacZ or Ad-Sirt6 and treated with10 μM atorvastatin or rosuvastatin for 6 h for Western blotting analysis. Values are means ± SEM. *, $p<0.05$ and **, $p<0.01$ versus vehicle. V, vehicle; A, atorvastatin; R, rosuvastatin.

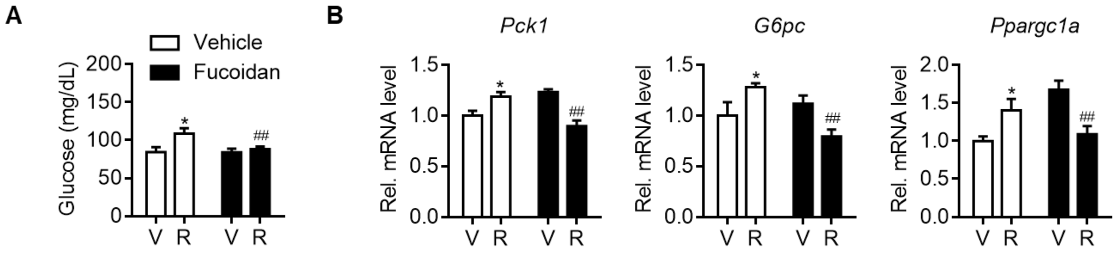

Figure S8. Effect of fucoidan on simvastatin induced hyperglycemia. (A) C57BL/6 mice were administered fucoidan (15 mg/kg) via oral gavage in combination with vehicle or rosuvastatin intraperitoneally for three days. Fast blood glucose was measured (n = 5). **(B)** mRNA levels of gluconeogenic genes were measured in mice. Values are means ± SEM. *, p<0.05 versus vehicle; $^{\#\#}$, p<0.01 versus rosuvastatin alone. V, vehicle; R, rosuvastatin.
